# Mating system of monoecious *Araucaria angustifolia* (Bert.) O. Kuntze, a predominately dioecious conifer species

**DOI:** 10.1186/1753-6561-8-S4-P103

**Published:** 2014-10-01

**Authors:** Moeses Andrigo Danner, Juliana Zanetti Ribeiro, Flávio Zanette, Juliana Vitória Messias Bittencourt, Alexandre Magno Sebbenn

**Affiliations:** 1 Universidade Tecnológica Federal do Paraná, Dois Vizinhos, Brazil; 2 Universidade Federal do Paraná, Curitiba, Brazil; 3 Universidade Tecnológica Federal do Paraná, Ponta Grossa, Brazil; 4 Instituto Florestal de São Paulo, São Paulo, Brazil

## Background

*Araucaria angustifolia* (Bert.) O. Kuntze is a mainly dioecious species that reproduces through outcrossing. However, some monoecious trees have been identified and they may reproduce through self-fertilization. The objective of this study was to confirm the expected relatedness of self-sibs of hand self-pollinated monoecious seed trees, and to investigate the mating system of open-pollinated progenies of monoecious *A. angustifolia* trees.

## Methods

To obtain the *A. angustifolia* progenies, we collected seeds from open-pollinated trees from three monoecious trees (MN_Ara, MN_SD and MN_Gua) and selfing was induced by hand-pollinating the monoecious tree PF_3. The seedlings of each progeny were labeled and needles were collected from 18 offspring from each progenies. Samples were lyophilized and the isolation of DNA followed the protocol described in [1]. Eight microsatellite loci (CRCAc1, As90, Ag20, Ag23, Ag45, Aang01, Aang14 and Aang28) were used to genotype the samples. Polymorphism was detected by labeling primers and PCR multiplex with fluorescent dyes, followed by fragment detection by capillary electrophoresis in automated sequencer. The size of the fragments (alleles) was determined and was estimated the mating system parameters using the MLTR software [2].

## Results and conclusions

Our results show that the selfed progenies (PF_3 × PF_3) are unquestionably the result of selfing (*t_m_* = 0.00 ± 0.00, *r_s_* = 0.99 ± 0.01, *P_SS_*= 1, Θ = 0.5, *N_e_*= 1) and all offspring are self-sibs, confirming the efficacy of induced selfing through hand-pollination of a monoecious tree. The three open-pollinated progenies of monoecious trees showed high multi-locus outcrossing rate (*t_m_*) values, but the values were significantly lower than unity (0.94 ± 0.01 and 0.95 ± 0.01), suggesting that some selfing did occur. The multilocus paternity correlation (*r_p(m)_*) of monoecious trees ranged from 0.10 to 0.29 and the progeny from MN_Ara produced values significantly higher than the two other progenies. The effective number of pollen donors (*N_ep_*) of these progenies ranged from 3.4 to 9.8, the coancestry coefficient within progenies (Θ) ranged from 0.152 to 0.172 and variance effective size ranged from 2.63 to 2.92. The open-pollinated progenies of monoecious seed trees were mainly half-sibs (*P_HS_* ranged from 0.63 to 0.79). Thus, the monoecious trees are reproductive mode by xenogamy, generating progeny with low proportion of self-sibs. The lower number of androstrobilus compared to ginostrobilus, as was observed in the monoecious tree PF_3, beyound heterozygous embryo selection in early stages, which occurs in *A. angustifolia* seeds [3], may explain the high outcrossing rate. Our results also suggest that monoecious trees have limited potential to modify the genetic structure through selfed seed production due to the very low estimated selfing rate and the rare occurrence of these trees in natural populations.
